# A Hypomorphic Vasopressin Allele Prevents Anxiety-Related Behavior

**DOI:** 10.1371/journal.pone.0005129

**Published:** 2009-04-09

**Authors:** Mirjam Bunck, Ludwig Czibere, Charlotte Horvath, Cornelia Graf, Elisabeth Frank, Melanie S. Keßler, Chris Murgatroyd, Bertram Müller-Myhsok, Mariya Gonik, Peter Weber, Benno Pütz, Patrik Muigg, Markus Panhuysen, Nicolas Singewald, Thomas Bettecken, Jan M. Deussing, Florian Holsboer, Dietmar Spengler, Rainer Landgraf

**Affiliations:** 1 Max Planck Institute of Psychiatry, Munich, Germany; 2 University of Innsbruck, Innsbruck, Austria; 3 Affectis Pharmaceuticals, Martinsried, Germany; 4 Helmholtz Zentrum, Institute of Developmental Genetics, Munich/Neuherberg, Germany; University of Queensland, Australia

## Abstract

**Background:**

To investigate neurobiological correlates of trait anxiety, CD1 mice were selectively bred for extremes in anxiety-related behavior, with high (HAB) and low (LAB) anxiety-related behavior mice additionally differing in behavioral tests reflecting depression-like behavior.

**Methodology/ Principal Findings:**

In this study, microarray analysis, *in situ* hybridization, quantitative real-time PCR and immunohistochemistry revealed decreased expression of the vasopressin gene (*Avp*) in the hypothalamic paraventricular (PVN) and supraoptic (SON) nuclei of adult LAB mice compared to HAB, NAB (normal anxiety-related behavior) and HABxLAB F1 intercross controls, without detecting differences in receptor expression or density. By sequencing the regions 2.5 kbp up- and downstream of the *Avp* gene locus, we could identify several polymorphic loci, differing between the HAB and LAB lines. In the gene promoter, a deletion of twelve bp Δ(−2180–2191) is particularly likely to contribute to the reduced *Avp* expression detected in LAB animals under basal conditions. Indeed, allele-specific transcription analysis of F1 animals revealed a hypomorphic LAB-specific *Avp* allele with a reduced transcription rate by 75% compared to the HAB-specific allele, thus explaining line-specific *Avp* expression profiles and phenotypic features. Accordingly, intra-PVN *Avp* mRNA levels were found to correlate with anxiety-related and depression-like behaviors. In addition to this correlative evidence, a significant, though moderate, genotype/phenotype association was demonstrated in 258 male mice of a freely-segregating F2 panel, suggesting a causal contribution of the *Avp* promoter deletion to anxiety-related behavior.

**Discussion:**

Thus, the identification of polymorphisms in the *Avp* gene promoter explains gene expression differences in association with the observed phenotype, thus further strengthening the concept of the critical involvement of centrally released AVP in trait anxiety.

## Introduction

Despite decades of intensive research, anxiety and depression disorders remain two of the most common and debilitating psychiatric illnesses. Their causes and appropriate medication are still elusive [1], and the number of affected people continues to increase [2], [3]. Depression is a highly heritable disorder; epidemiologic studies have shown that about 40–50% of the risk for depression is genetically determined [4], [5] and that depression is in 60% of all cases comorbid with anxiety disorders [6], [7]. These findings are strongly supported by familial and twin studies [8]. However, the considerable evidence for heritability has not been accompanied by satisfactory progress towards identifying genetic risk factors underlying anxiety and depression. An improved understanding of the genetic contributions would have significant implications for the diagnosis and treatment of neuropsychiatric disorders.

In order to identify shared genotype/phenotype relationships in animals and humans, the same gene should affect analogous phenotypes in both species. Thus, a useful approach should depend on conserved gene function, the presence of functional polymorphisms in this gene in both species and the choice of an appropriate analogous phenotype in both the model species and in humans [9]. The gene for the nonapeptide vasopressin (AVP) fulfills all these requirements. It is evolutionary conserved as both antidiuretic hormone after peripheral secretion and anxiogenic stress-related neuropeptide after central release [10]–[12]. Moreover, the presence of functional polymorphisms and subsequent deficits in expression/processing in rodents and patients leads to signs of both central diabetes insipidus and emotional alterations, e.g. decreased anxiety-related behavior [13]–[16]. Finally, trait anxiety with the genetic predisposition towards either high (HAB) or low (LAB) anxiety-related behavior is a heuristically and clinically relevant phenotype, with a high degree of face, construct and predictive validity [17].

Preclinical studies, including findings from the HAB/LAB rat model [18]–[21] and voles [22], as well as clinical observations [23] support a direct involvement of centrally released AVP in anxiety/depression-like behaviors and disorders. To make use of the advantages of the full spectrum of molecular-genetic approaches, CD1 mice were selectively inbred for either low or high anxiety-related behavior in analogy to the HAB/LAB rat model, while maintaining a high degree of similarity in non-selected traits. Along with their divergence in anxiety-related behavior, which is reflected in a variety of distinct behavioral paradigms, these mouse lines also exhibit differences in tests of other metrics, including depression-like and explorative behaviors [24]. The final goal of our selective breeding strategy is to identify candidate genes of anxiety and to prove their causal involvement in the anxiety phenotype, while simultaneously reducing the possible influence of genetic drift and the concomitant risk of indicating false associations between the gene of interest and a given phenotype, a risk particularly high in inbred lines or strains. Therefore, a series of measures have been employed in order to minimize the risk related to genetic drift: (i) in each selectively bred line, several independent sublines are run in parallel; (ii) identified genetic polymorphisms are tested in a freely-segregating F2 panel and are accepted to be causal if they co-segregate with the phenotype; (iii) in addition to the divergent lines, normal anxiety (NAB) mice are used to represent intermediate anxiety-related behavior; and (iv) replication is important in terms of the impact of founder effects and genetic drift and should be run concurrently with the main breeding protocol in the same or another species, thus also facilitating complementary inter-species genetics [9].

Therefore, if *Avp* gene expression is causally related to the HAB vs. LAB phenotype, genetic polymorphisms contributing to this phenomenon should be detectable not only in selectively bred rats but also in mice. Indeed, LAB mice display a deficit in hypothalamic AVP [25]. In light of the functional impact of polymorphisms in the *Avp* regulatory regions for both gene expression and anxiety-related behavior in rats [18], we here addressed the question whether polymorphisms in the *Avp* promoter of LAB mice may be identified and contribute to the neuroendocrine and behavioral divergence demonstrated in HAB vs. LAB mice. This will provide new insights into the genetic predisposition that shapes emotionality in both inter-individual variation and psychopathology.

## Materials and Methods

### Animals

All animals tested were bred in the animal facilities of the Max Planck Institute of Psychiatry in Munich (Germany) as described previously [24]. Briefly, >250 animals from >25 litters of outbred Swiss CD1 mice purchased from Charles River (Sulzfeld, Germany) were used as a starting point for selective and bidirectional breeding for anxiety-related behavior on the elevated plus-maze (EPM) at the age of seven weeks. Males and females that spent either the least or most time on the open arms of the EPM were mated to establish the HAB and LAB lines, respectively. This intra-strain approach was chosen to ensure that the HAB/LAB lines show a maximum divergence in the selected trait, while maintaining a high degree of similarity in non-selected traits. The animals were routinely tested at the age of seven weeks, with HAB mice spending <10% and LAB >50% of the total test time on the open arms of the EPM. NAB mice were bred for intermediate anxiety-related behavior. As >80% of CD1 mice spend between 25% and 35% of their time on the open arms of the EPM, this range was chosen for the selection of NAB mice without any overlapping either with LAB or with HAB animals. While CD1 mice of the parental generation were used as NAB controls by Krömer *et al.* [24] and Kessler *et al.* [25], we then decided to start inbreeding them in parallel with HAB and LAB mice, aiming at further reducing all variables that are unrelated to anxiety, such as slight differences in body weight between outbred vs. inbred animals.

All experiments were carried out on adult male or female HAB, LAB, NAB, HABxLAB F1 intercross and F2 mice. In the present study, HAB and LAB mice of generations 12–23 and NAB mice of generation 1 were used. Importantly, in a wide variety of tests and parameters, the scores of NAB and F1 intercross controls were found to be similar, if not identical, and between the scores of HAB and LAB mice ([24]; unpublished results).

The animal studies were both approved by local authorities and conducted according to current regulations for animal experimentation in Germany and the European Union (European Communities Council Directive 86/609/EEC).

### EPM test

The EPM consisted of a plus-shaped platform elevated 37 cm above the floor, with two open (30×5 cm), two closed (30×5×15 cm) arms and a connecting central zone (5×5 cm) made of grey PVC. The open arms were lit by white light of 300 lux intensity, the neutral zone of 60 lux intensity and the closed arms of 5 lux intensity. The maze was cleaned with water containing a detergent and dried before each trial.

Animals were directly transferred from their homecage to the test apparatus, starting with the animal placed on the central part facing one closed arm. During the 5-min exposure, various parameters were recorded by means of a video/computer system. Analysis was performed *post hoc* by an observer blind to the line using Plus-maze V2.0 (Ernst Fricke, Munich, Germany) [24], [26].

### Tail-suspension test

In the tail-suspension test, animals were suspended at the end of their tail to a bar at a height of 37 cm above the floor with an adhesive tape [27]. Four animals were tested in parallel during each test. The behavior was videotaped and the duration of total immobility (immobility of all four extremities) scored by an observer blind to the line using Eventlog 1.0 (EMCO Software, Reykjavik, Iceland).

### Forced swim test

In the forced swim test each mouse was placed for 6 min into a glass cylinder (height: 23 cm; diameter: 11 cm) containing 15 cm of water at 22–23°C. For each videotaped trial, immobility time (floating) was analyzed by an observer blind to the line using Eventlog 1.0 (EMCO Software). A mouse was judged immobile when it stopped any slight movements, except those that are necessary to keep its head above water [28].

### Open field

The round open field, having a diameter of 60 cm, was made of black and grey PVC and was optically divided into an inner (diameter: 30 cm) and an outer area to allow quantification of the time spent in each compartment. To devise the test situation as less aversive as possible and to make the test suitable to measure locomotor activity independent of anxiety, the arena was illuminated with dim light of 60 lux. Before each trial, the chamber was cleaned with water containing a detergent. The animals' behavior was videotaped during the 5-min test time and the parameter path length was analyzed with the tracking software ANY-maze version 4.30 (Stoelting, Wood Dale, IL, USA).

### Gene and peptide expression profiling

If not indicated otherwise, all animals used for gene and peptide expression profiling or receptor radioautography were sacrificed by decapitation under isoflurane anesthesia (Abbott, Wiesbaden, Germany), between 9a.m. and 12p.m. The brains were removed, snap-frozen in N-methylbutane (Carl Roth, Karlsruhe, Germany) and stored at −20°C or −80°C.

#### Tissue sampling for microarray and quantitative real-time PCR

The tissue micropunching technique [29] was applied for acquiring tissue samples from specific regions with micropunchers of 0.5 mm and 1 mm diameter (Fine Science Tools GmbH, Heidelberg, Germany). Punches were collected from 200 µm sections between −0.56 mm and −0.96 mm from bregma, medially 0.8 mm above the ventral tissue edge around the dorsal end of the 3^rd^ ventricle to obtain the PVN (Ø = 1 mm) and bilaterally from the optic tract (Ø = 0.5 mm) in order to acquire tissue from the SON. All coordinates were based on the mouse brain atlas [30]. Tissue punches were kept at −80°C until further processing.

#### RNA extraction and reverse transcription from tissue punches

Tissue punches were homogenized with a pipette in 300 µl Trizol (Tri Reagent, Sigma-Aldrich). Then 30 µl bidistilled water, 1 µl linear acrylamide (5 mg/ml, Ambion, Austin, TX) and 60 µl chloroform were added before continuing RNA extraction with a standard protocol, using n-propanol for RNA precipitation overnight. The yield of total RNA was 0.3–1.5 µg.

All isolated RNA was further processed for microarray analysis using the Ambion Amino Allyl Message Amp aRNA kit (Ambion) in two rounds of amplification. For qPCR, not more than 1 µg of total RNA was reverse transcribed with Superscript II (Invitrogen, Karlsruhe, Germany) after DNAse treatment. All steps required for reverse transcription were performed according to the manufacturer's protocol. For quality control, a small aliquot of cDNA was analyzed on an agarose gel.

#### Microarray analysis

Whole-genome gene expression data were acquired using a dual-color experimental design (cyanine 3 and cyanine 5) on an in-house microarray platform (MPI24K) with pooled samples (six LAB vs. six HAB male mouse brain pools) using ten technical replicates including a dye-swap of the hybridized samples on five microarray slides. Pretreatment of microarray probes, assay hybridization, scanning, data evaluation and statistical analysis were performed as described previously [31], [32]. Briefly, microarray slides were scanned in a PerkinElmer ScanArray 4000 (PerkinElmer Life and Analytical Sciences, Shelton, CT) and quantified using QuantArray software (GSI Lumonics, Billerica, MA) with a fixed-circle quantification protocol. Data normalization was performed in multiple steps based on the raw fluorescence intensities ensuring the elimination of artifacts resulting from the unequal distribution of probes or from unbalanced fluorescence intensities within one microarray slide.

#### Quantitative PCR analysis

cDNA of male or female, LAB, HAB or NAB mice was analyzed by quantitative real-time PCR (qPCR), using the LightCycler® FastStart DNA MasterPLUS SYBR Green I reagent (Roche Diagnostics, Mannheim, Germany) or the QuantiFast SYBR Green PCR Kit (Qiagen, Hilden, Germany) according to the manufacturer's instructions and the respective oligonucleotide primers for *Avp*, *Avpr1b*, *Oxtr*. For reaction conditions and primer sequences see Table 1. Experiments were performed in duplicates with the Lightcycler®2.0 instrument (Roche Diagnostics) under the following PCR conditions: initial denaturation at 95°C for 10 min, then 40 cycles of denaturation (95°C for 10 s), annealing (56–65°C for 4–5 s) and elongation (72°C, 7–13 s) for the Roche-kit. Similar conditions were applied for the Qiagen-kit, only the annealing and extension phase were standardly substituted by a combined annealing/extension phase at 60°C for 30 s. At the end of every run, a melting curve (50–95°C with 0.1°C/s) was generated in order to ensure the homogenity of the PCR product.

**Table 1:** Forward and reverse primers used for quantitative PCR and their specific annealing temperatures.

Gene symbol	Orientation	Primer sequence (5′→3′)	qPCR assay	Annealing temp. [°C]
*Avpr1b*	forward	GCCCCTAATGAAGATTCTACCAATGTGG	2	
	reverse	CTGCAGCAGGCGCGGTGACT		
*Oxtr*	forward	CAGTGAAGATGACCTTCATC	2	
	reverse	CCTTCAGGTACCGAGCAGAG		
*Avp*	forward	TCGCCAGGATGCTCAACAC	1	56
	reverse	TCCGAAGCAGCGTCTTGG		
*Gapdh*	forward	CCATCACCATCTTCCAGGAGCGAG	1, 2	65
	reverse	GATGGCATGGACTGTGGTCATGAG		
*Hprt1*	forward	GTCAAGGGCATATCCAACAACAAAC	1, 2	57
	reverse	CCTGCTGGATTACATTAAAGCACTG		
*Atp2b1*	forward	CATTACGGAAAATACAGGAGAGC	1, 2	62
	reverse	TGCTTCCCAGACTAACTGAAGAA		

Numbers for qPCR assay indicate the SYBR Green Kit used (1: Roche; 2: Qiagen).

Crossing points were calculated by the LightCycler®Software 4.0 (Roche Diagnostics) using the absolute quantification fit points method. Threshold and noise band were set to the same level in all compared runs.

Relative gene expression was determined by the 2^−ΔΔCT^ method [33] using the real PCR efficiency calculated from an external standard curve. The crossing point was normalized to the housekeeping genes *Gapdh*, *Hprt* and *Atp2b1* (Table 1), respectively and values were calculated relative to the expression mean of LAB mice.

#### Avp and Oxytocin mRNA in situ hybridization

Brains were sectioned into 14 µm slices with a cryostat (Microm HM 500, Microm, Walldorf, Germany). Three sets of sections at PVN level were taken and used for *Avp* or Oxytocin (*Oxt*) *in situ* hybridization (ISH) according to established protocols [20], [34].

Briefly, sections were dehydrated in increasing ethanol concentrations, delipidated with chloroform, rinsed in ethanol and air-dried afterwards. A highly specific 48-bp oligonucleotide sequence directed against the last 16 amino acid coding region of the glycoprotein that AVP does not share with OXT (5′ GGG CTT GGC AGA ATC CAC GGA CTC TTG TGT CCC AGC CAG CTG TAC CAG 3′; [35], [36]) was used for hybridization with *Avp*. Detection of *Oxt* mRNA was performed by hybridization of a similarly specific 48-bp oligonucleotide (5′ CTC GGA GAA GGC AGA CTC AGG GTC GCA GGC GGG GTC GGT GCG GCA GCC 3′; [35]). The oligonucleotides were labeled by using [^35^S]ATP (NEN DuPont, Bad Homburg, Germany) and terminal transferase (Tdt, Boehringer, Mannheim, Germany) and purified by tRNA (Sigma, Germany) precipitation. Tissue sections (5 sections per slide) were saturated with 100 µl of hybridization buffer (deionized formamide, 20×SSC, dextran sulfate, 0.2 M Na-phosphate buffer, Dehnhard's solution, 20% sarcosyl, salmon sperm DNA, 5 M DTT (DL-Dithiothreitol)) containing 10^6^ cpm/100 µl for the *Avp*-^35^S and 0.5×10^6^ cpm/100 µl for the *Oxt*-^35^S oligoprobe. Coverslipped sections were incubated in a moist chamber for 18–22 h at 45°C. After several washes in 1×SSC, the sections were dehydrated and air-dried [20]. For all following steps see section of data analysis below.

#### AVP immunohistochemistry in the PVN

Immunohistochemistry was performed as described previously [37]. In brief, animals were deeply anaethesized by an overdose of sodium pentobarbital (200 mg/kg) by i.p. injection prior to cardiac perfusion. Brains of these unstressed mice were removed and postfixed at 4°C in 4% paraformaldehyd over night and then stored in 0.2 M phosphate buffer until sectioning. Immunofluorescence staining was used to visualize AVP. Vibratome (Series 1000, Ted Pella Inc., Redding, CA) sections (50 µm) were preincubated in immunobuffer containing 5% normal donkey serum (Jackson ImmunoResearch, West Grove, PA) and then incubated for 48 h at room temperature with a polyclonal rabbit antibody to AVP (dilution 1∶10000, Peninsula Laboratories, Belmont, CA). Sections were exposed for 2.5 h to a Cy2-conjugated donkey anti-rabbit IgG (dilution 1∶100, Jackson ImmunoResearch). Negative controls were performed by omission of the primary antibodies. No staining was observed in any of the control sections. Images were recorded on a fluorescence microscope (Olympus BX-51, Vienna, Austria), equipped with a U-M41001 filter system (excitation filter, 455–495 nm; dichromatic mirror, 505 nm; emission filter, 510–555 nm) for Cy2 (excitation maximum 492 nm; emission maximum 510 nm), using a digital camera (Olympus DP50) and analySIS® image processing software (Soft Imaging Systems, Münster, Germany).

#### AVP receptor 1a autoradiography

For AVP receptor 1a (V1a) autoradiography, frozen cryostat brain sections (14 µm) of HAB and LAB mice were utilized and processed as described elsewhere [20]. Before incubation, the slides were fixed using 0.1% PFA and washed in Tris buffer. For competitive displacement of endogenous AVP and for receptor binding, slides were incubated (60 min, RT) with the AVP V1a-R antagonist ^125^I-lin AVP (NEX310, NEN DuPont, Boston, MA; 200 cpm/µl). After several washes, the slides were air-dried.

#### Data analyses

For all ISHs and receptor binding analyses, the sections were exposed to a Kodak BioMax film (Amersham, Braunschweig, Germany) for 1–3 days (increased exposure time for *Oxt* due to the lower amount of labelled oligoprobe). Autoradiographs were quantified using an image analysis optical software (Optimas 5.2, Optimas Corporation, Seattle, WA). Autoradiograms from ISH and receptor binding studies were analyzed using the optical density readings (grey intensity). Three to six sections were analyzed and the highest expression (measured as hybridization signal minus background) per animal was taken for calculating mean hybridization per nucleus and line [20]. Due to the high expression of *Avp* and *Oxt* mRNAs in the hypothalamic PVN and SON, we focused on these nuclei in LAB, HAB, NAB or F1 animals. For V1a receptor autoradiography, the amygdala, bed nucleus of the *stria terminalis*, hippocampus, lateral septum, PVN, SON, suprachiasmatic nucleus and ventral pallidum were analyzed.

### *Avp* sequencing

Genomic DNA was purified from tail tissue clips of HAB and LAB mice from the 20^th^ generation, using the NucleoSpin Tissue kit (Macherey-Nagel, Düren, Germany). The *Avp* coding sequence [25] as well as 3 kbp up- and downstream the coding region were amplified using 12 primer pairs (MWG Biotech, Ebersberg, Germany; Table 2). All PCR amplifications were carried out by using *Taq* polymerase (Fermentas, St. Leon-Rot, Germany) in 25-µl reactions under the following conditions: initial denaturation at 94°C for 4 min, 40 cycles of denaturation (94°C for 1 min), annealing (52–56°C for 1 min) and extension (72°C for 1 min), concluded by a final extension of 10 min at 72°C. Purification of the PCR products was performed with the *NucleoFast* kit (Macherey-Nagel). Then, the PCR products were ligated into the pGEM-T vector (Promega Corp., Madison, WI), according to the manufacturer's instructions and transformed into competent *E. coli* DH5α cells via electroporation. Transformed bacteria were collected in 1 ml SOB medium, 150 µl of the suspension were incubated overnight at 37°C on lysogeny broth/ampicillin agar plates inoculated with 5-bromo-4-chloro-3-indolyl-β-D-galactoside (X-Gal: 25 µl; 10%) and isopropyl-β-D-thiogalactopyranoside (IPTG: 30 µl; 0.1 M) solved in dimethyl sulfoxide. Colonies were picked and their plasmid inserts were amplified using T7 and SP6 primers in a 35- cycles PCR with an annealing temperature of 49°C and otherwise identical conditions, as described above. 5 µl of each PCR product were analyzed on a 1.5% agarose gel, another 2 µl were purified with the NucleoFast 96 PCR kit (Macherey-Nagel) and used for the sequencing reaction (BigDye Terminator Kit, Applied Biosystems, Foster City, CA) that was performed according to the manufacturer's instructions. Sequencing reaction products were purified with the Sequencing Reaction Cleanup Kit (Millipore, Billerica, MA) and resolved by electrophoresis on a 3730 DNA Analyzer (Applied Biosystems). DNA sequences around the identified polymorphic sites were analyzed via the Transcription Element Search System (TESS, http://www.cbil.upenn.edu/cgi-bin/tess/tess). Putative binding sites of transcription factors were additionally checked for their function and occurrence within the brain according to the NCBI database (http://www.ncbi.nlm.nih.gov/).

**Table 2:** Forward and reverse primers used for Avp sequencing and their specific annealing temperatures.

No.	Forward primers (5′→3′)	Reverse primers (5′→3′)	Annealing temp. [°C]
01	GACACAGTGTGCCTCTATG	GCTCTCCTGGACCTTCTG 3′	56
02	AATACTCTAGGAAGAAGACAA	GAAACAGCTTCCTGGTCA 3′	56
03	GGACATGCCACTCAAGGG	TACAGGCGTGCATCACGG 3′	56
04	CTAGAAGCCGTGGGCTAGGT	GGTGGGGAGAGCTTGGGAATAG	52
05	CATTGCCACCATAGCTTTC	CTCTTGGGCAGTTCTGGAAG	52
06	GAGCAGAGCCTGAGCTGCACACAGT	ACATACAATACAACAGATCTG	56
07	CCATGCCCAAGTGGAGC	GCTGGAACGAGGCCAAG	56
08	AAAGCAGCAGGTGACACTAGG	CTATGCACGACTTCGGGTGTG	55
09	ACTCCGTGGATTCTGCCAAGC	GATGCCTTCTGCTCCTGAGAC	55
10	GCACGGAAATAGACAAGATAG	AACTGACCATCCTGAGCCACC	55
11	AGAGATTAGTCTCAGTGACCTG	CTGGAGTTGTGAGGTGGTTGTG	55
12	ACGGCTCAAGGAGGTAGGCG	AAGTGACCACAAAGCACGGAG	55

### Allele-specific transcription analysis

PVN and SON were dissected from the hypothalamus of four female F1 mice that were heterozygous for the recently described C(40)T (rs50049109) single-nucleotide polymorphism (SNP) [25]. Total RNA was extracted using a Trizol (Invitrogen) chloroform extraction protocol and reverse transcribed with Omniscript (Qiagen GmbH, Hilden, Germany). 208 bp of the *Avp* transcript containing the SNP at position C(40)T were amplified by PCR in 40 identical cycles of denaturation at 94°C for 1 min, annealing at 56°C for 1 min and elongation at 72°C for 1 min using *Taq* polymerase (Fermentas) and 5′ GAG CAG AGC CTG AGC TGC ACA CAG T 3′ as forward and 5′ AGC AGA TGC TTG GTC CGA AGC ACG 3′ as reverse primers (MWG Biotech). Amplified fragments were ligated into a vector and transfected into competent *E. coli* cells that were grown overnight as described above.

From each agar plate, 25 colonies containing insert DNA (according to white/blue selection) were picked and sequenced. The specific alleles from each transcript were determined by the C(40)T polymorphism.

### *Avp* promoter deletion and signal peptide polymorphism in a freely-segregating F2 panel

Biallelic DNA sequence polymorphisms were assessed using a custom-designed oligonucleotide pool and the GoldenGate assay on an Illumina Sentrix Array Matrix platform (Illumina, San Diego, CA). In the parental generation, the two polymorphisms located in the coding sequence or the promoter of *Avp* display opposite homozygosity in HAB (+/+ and CC) vs. LAB (−/− and TT) animals. A total of 508 F2 offspring of a HABxLAB F1 intercross progeny were phenotyped and genotyped at these two loci based on DNA isolated from tail tips as described recently [25]. Genotyping was performed using the manufacturer's protocol and genotypes were determined using the Illumina BeadStudio software 3.1.0.0 and the genotyping module 3.1.12. Twenty four samples were genotyped twice, assuring data quality and reproducibility.

### Statistical analyses

The presented data are given as means+SEM and were statistically analyzed using SPSS 12.0. Two independent groups were compared using the Mann-Whitney U-test. For three independent groups, the nonparametric Kruskal-Wallis H-test was used with *post hoc* Mann-Whitney U-test comparisons, including sequential Bonferroni correction of the respective p-values, if applicable. For correlation analysis, a Spearman-Rho correlation analysis was performed. For allele-specific transcription analysis, deviation from the expected value was estimated using the chi-square test.

For the F2 panel, association analysis was performed using a permutation approach (WG-Permer, www.mpipsykl.mpg.de/wg-permer). Trait values were rank-transformed to protect against artifacts. Analysis was done using a genotypic model, i.e. the three genotypic classes possible for each of the phenotypes were treated as a separate class each and global test on equality of the three means was performed.

## Results

### Behavioral phenotype and *Avp* expression in LAB, HAB and NAB mice

#### EPM

**Figure 1:**
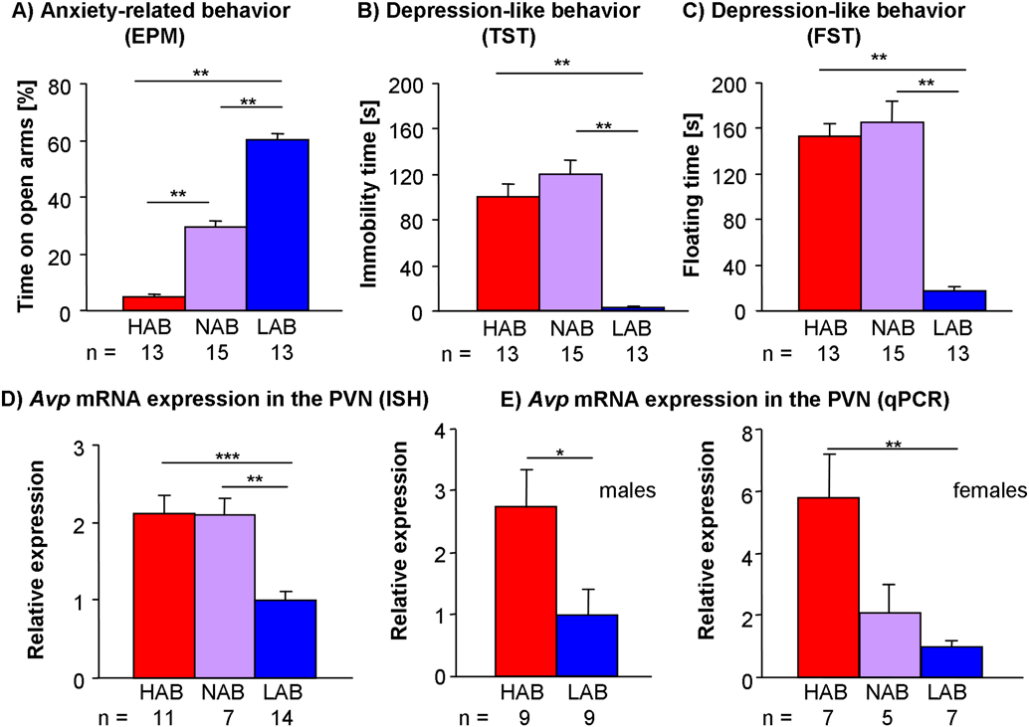
Alterations in behavior and vasopressin *(Avp)* expression. Phenotypic measures of HAB/NAB/LAB mice reflecting behavior (A) on the elevated plus-maze (EPM), in the (B) tail-suspension test (TST) and (C) forced swim test (FST) and corresponding vasopressin (*Avp*) expression patterns as measured by (D) *in situ* hybridization (ISH) in the paraventricular nucleus (PVN) in male mice and by (E) quantitative PCR (qPCR) in the PVN of male and female mice. Data are shown as means+SEM; * p<0.05; ** p<0.01; *** p<0.001; Mann-Whitney-U test.

In accordance with our breeding strategy, LAB, HAB and NAB male mice showed significant differences in the percent of total test time spent on the open arms of the EPM (Fig. 1A), with LABs spending most time (about 60%) on the open arms, HABs least time (about 5%) and NAB animals displaying an intermediate phenotype (about 30%; p<0.01 for all group comparisons).

#### Tail suspension and forced swim test

Male LAB animals exhibited a dramatically decreased immobility in the tail suspension test (Fig. 1B) (p<0.01 vs. HAB and NAB). Similarly, LAB mice showed decreased floating time in the forced swim test relative to HAB and NAB mice (p<0.01 each; Fig. 1C).

### Gene and protein expression analysis and validation

Analysis of genome wide expression revealed a significantly decreased *Avp* mRNA level in the PVN (p<0.05) and showed a tendency towards the same direction in the SON (p<0.1) of LAB compared to HAB mice. Importantly, no differences were observed for *Avpr1a*, *Avpr1b*, *Oxt or Oxtr* (p>0.1) in the respective brain regions investigated (Table 3).

**Table 3:** Regulation of gene products of the vasopressin and oxytocin systems from the MPI24K platform-based microarray experiment of HAB vs. LAB mice.

Gene symbol	PVN	SON
*Avp*	T58.1	T53.2 ^1^
*Avpr1a*	T95.2 ^2^	105.0 ^2^
*Avpr1b*	105.0	100.0 ^3^
*Oxt*	T82.0	T80.0 ^3^
*Oxtr*	100.0	102.0 ^3^

Values indicate the percentage of gene expression in LAB compared to HAB mice with HAB = 100%. Superscript numbers indicate the validation status: (1) significant gene expression differences confirmed by both quantitative PCR (qPCR) and *in situ* hybridization; (2) no differences in gene expression confirmed by receptor autoradiography; (3) no differences in gene expression confirmed by qPCR and *in situ* hybridization, respectively.

**Figure 2:**
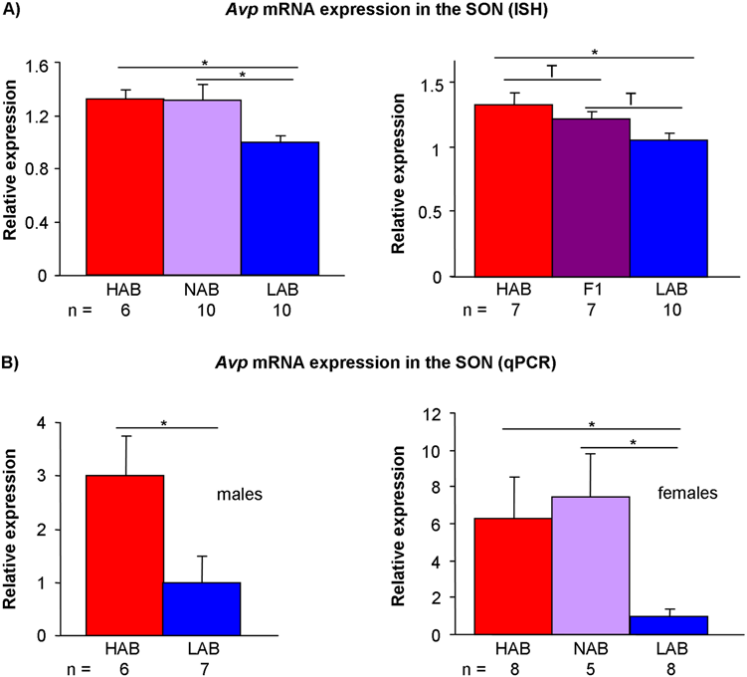
Gene expression of HAB, NAB, LAB, and HABxLAB F1 intercross mice. Vasopressin (*Avp*) mRNA expression in the supraoptic nucleus (SON) measured by (A) *in situ* hybridization (ISH) and (B) quantitative PCR (qPCR) under basal conditions in male and female mice. Data are shown as means+SEM; T p<0.1; * p<0.05; ** p<0.01; Mann-Whitney-U test.

**Figure 3:**
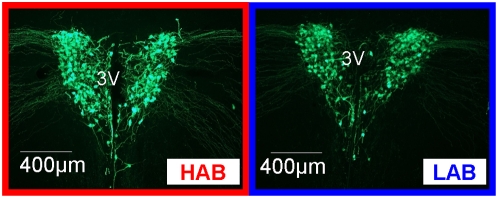
Vasopressin (AVP) immunohistochemistry analysis. AVP fluorescence antibody-staining of the paraventricular nucleus (PVN) flanking the 3rd ventricle (3V) in HAB and LAB mice. For corresponding *in situ* hybridization, see Fig. 7.

Confirming microarray data, ISH revealed a decreased expression of *Avp* in both the PVN (Fig. 1D) and SON (Fig. 2A) in male LAB compared to both HAB and NAB mice (PVN: p<0.01 vs. HAB and NAB; SON: p<0.05 vs. HAB and NAB). Similarly, qPCR analysis showed a reduced *Avp* expression in the PVN (Fig. 1E) and SON (Fig. 2B) of both male and female LABs compared to HAB or NAB animals (PVN of male mice: p<0.05 vs. HAB; SON of male mice: p<0.05 vs. HAB; PVN of female mice: p<0.01 vs. HAB; SON of female mice: p<0.05 vs. HAB and NAB). To address the question whether the described differences in AVP transcript levels actually translate into biologically relevant AVP peptide contents, we carried out immunohistochemistry with an antibody directed against AVP. Confirming ISH data, these experiments showed a decreased amount of AVP peptide in LAB compared to HAB mice in the PVN (Fig. 3).

**Figure 4:**
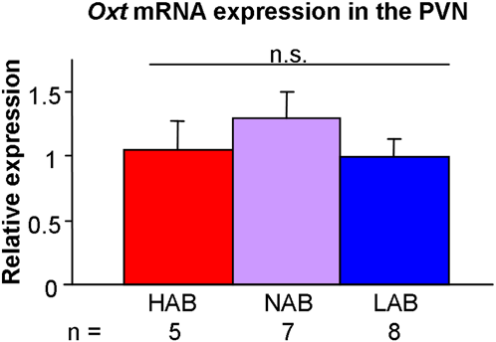
Oxytocin *(Oxt)* mRNA expression. *Oxt* in the paraventricular nucleus (PVN) measured by *in situ* hybridization under basal conditions in male HAB, NAB and LAB mice. Data are shown as means+SEM.

In contrast to AVP, the structurally related hypothalamic neuropeptide OXT failed to reveal any significant differences among the lines (Fig. 4) as detected by ISH. Also, no differences in the expression of *Avpr1b* or *Oxtr* (p>0.1) could be confirmed using qPCR (Table 3). Likewise, V1a autoradiography failed to reveal any difference between the HAB and LAB lines in a variety of brain regions critically involved in the regulation of anxiety- and depression-related behaviors, thus further strengthening the concept that events upstream of the receptor contribute to the behavioral phenotype of HAB and LAB mice (Table 3).

### Polymorphic AVP alleles

**Figure 5:**
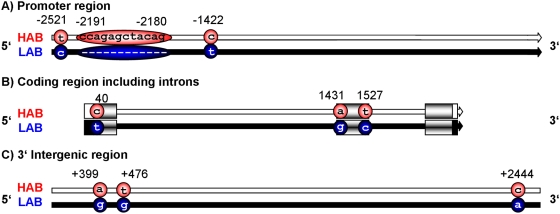
Vasopressin *(Avp)* gene sequence of HAB vs. LAB mice. Polymorphic sites are indicated with positions from transcription start (−1 to −2600 bp) in (A) the promoter region (two SNPs and deletion in LABs); (B) within the *Avp* coding sequence from transcription start (1 to 1960 bp; three SNPs); (C) in the intergenic region between *Avp* and oxytocin *(Oxt)* from the end of the last exon (+1 to +2600 bp; three SNPs). Exons and untranslated regions (UTRs) are indicated by boxes (exons shaded, UTRs completely filled black or white).

Next we investigated whether differences in AVP expression originate from differences in the sequence of HAB and LAB alleles that could underpin differences in the recruitment of proteins driving transcription. Sequencing of the *Avp* gene resulted in the identification of nine polymorphic sites, of which eight correspond to SNPs and one comprises a 12 bp deletion (Fig. 5).

The upstream promoter region contains two SNPs: T(−2521)C with LABs carrying cytosine and HABs thymine and C(−1422)T with LABs carrying thymine and HABs cytosine. Furthermore, LAB mice miss a 12 bp segment mapping to Δ(−2180–2191). The gene-coding locus contains three SNPs: C(40)T with LABs carrying thymine and HABs cytosine, A(1431)G with LABs carrying guanine and HABs adenine and T(1527)C with LABs carrying cytosine and HABs thymine (see also [25]). The downstream enhancer region, also referred to as the intergenic region between *Avp* and *Oxt*, contains three SNPs: A(+399)G with LABs carrying guanine and HABs adenine, T(+476)G with LABs carrying guanine and HABs thymine and C(+2444)A with LABs carrying adenine and HABs cytosine. A detailed *in silico* search for potential transcription factor binding sites at the polymorphic loci led to the identification of a number of candidates that are summarized in Table 4.

**Table 4:** Polymorphisms in the promoter and downstream enhancer region between the HAB and LAB specific sequence with probable binding factors.

Polymorphism	1st binding factor	2nd binding factor	Reference
T(−2521)C	NF-1	C/EBPalpha	(49,50)
Δ(−2180–2191)	NF-1	C/EBPbeta	(48,49)
C(−1422)T	—		
A(+399)G	c-Ets-2	C/EBPbeta	(51)
T(+476)G	AP-1 / GATA-1		
C(+2444)A	—		

In all our studies, involving LAB, HAB, NAB and F2 mice, the C(40)T SNP and 12 bp deletion were in complete linkage, i.e. the homozygous LAB-specific combination of alleles (TT) at the 40^th^ bp of the transcript-coding sequence was strictly associated with the absence of the 12 bps of the promoter. In analogy, if the transcript-coding alleles were CT or CC, the −2180–2191 locus bore the +/− and +/+ allelic constellation, respectively. The most frequent genotypic combination in NAB mice was the HAB-specific one (Table 5).

**Table 5:** Allele frequency in a NAB population (n = 165) for the SNP in the *Avp* signal peptide and the strictly linked promoter deletion.

Locus (40)	Frequency in NAB	Δ(−2191–2180)
CC	73.3%	+/+
CT	26.1%	+/−
TT	T0.6%	−/−

### Allele-specific *Avp* transcription in heterozygous HABxLAB F1 intercross mice

**Figure 6:**
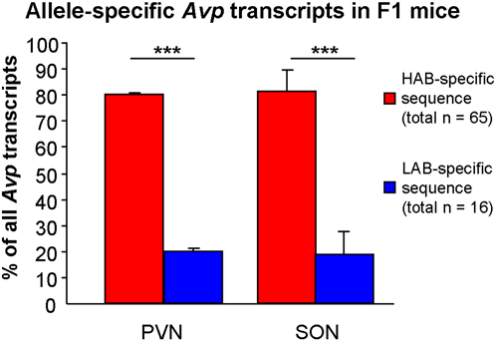
Allele-specific vasopressin *(Avp)* expression. Proportion of HAB allele-specific vs. LAB allele-specific *Avp* transcripts from heterozygous F1 (HABxLAB intercross) mice in the paraventricular nucleus (PVN; χ^2^ = 14.4 p = 1.4e-4) and the supraoptic nucleus (SON; χ^2^ = 15.2 p = 4.8e-5). Data are shown as means+SEM; *** p<0.001.

To obtain evidence for a functional role of these polymorphisms in contributing to different levels of AVP expression in male and female LAB vs. HAB mice, we studied the activity of each allele in an F1 intercross. Under this condition, both alleles are contained in the same cellular background, thus eliminating differential synaptic input as a confounding factor. In support of an allele-dependent effect, heterozygous F1 mice revealed intermediate levels of *Avp* mRNA expression in the SON in comparison to LABs and HABs (p<0.1 F1 vs. HAB and LAB, p<0.05 LAB vs. HAB; Fig. 2A). In line with these findings, the LAB-specific allele displayed a significantly decreased expression relative to the HAB-specific one in both the PVN and SON (p<0.001; Fig. 6), consistent with *Avp* mRNA expression data of HAB vs. LAB mice (see Figs. 1D, E; 2A, B).

### Correlation of *Avp* mRNA in the PVN and anxiety/depression-like behaviors of HAB, F1 and LAB mice

**Figure 7:**
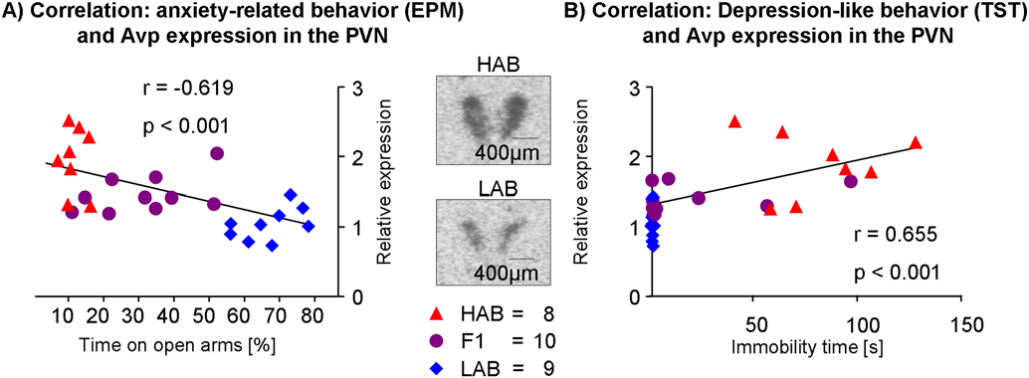
Correlation between gene expression and behavior. Correlation of vasopressin *(Avp)* mRNA expression in the paraventricular nucleus (PVN) with (A) anxiety-related behavior and (B) depression-like behavior of HAB, F1 and LAB male mice under basal conditions. For corresponding immunohistochemistry, see Fig. 3.

In light of the above data pointing to a dual role of hypothalamic AVP in contributing to HAB and LAB phenotypes, respectively, we performed correlation analyses with the aim to obtain a more detailed picture of AVP's role in anxiety- and depression-like behaviors. *Avp* mRNA expression in the PVN of male LAB, F1 and HAB mice under basal conditions is significantly correlated with anxiety-related behavior (r = 0.619; p<0.001; Fig. 7A) as well as depression-like behavior (r = 0.655; p<0.001; Fig. 7B).

### Association between the phenotype and polymorphisms at the AVP locus

**Figure 8:**
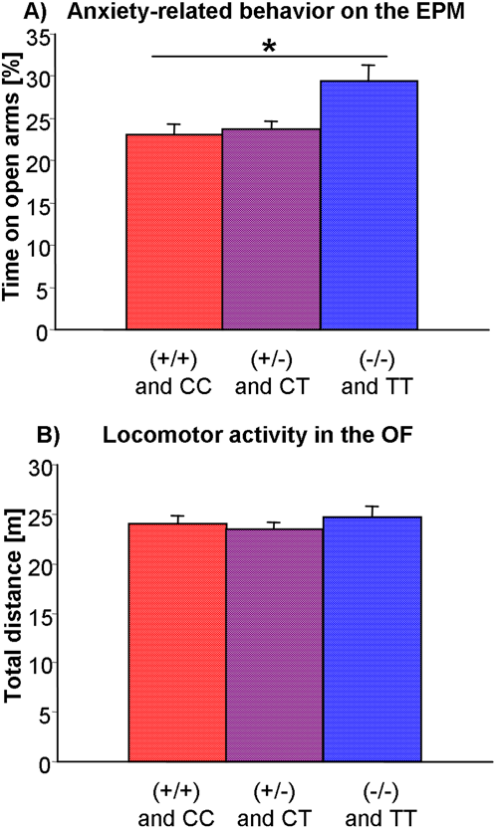
Association between anxiety-related behavior and the *Avp*-related genotypes in the freely-segregating F2 panel. Phenotypic indices of male F2 mice with HAB grandmothers and *Avp*-related genotypes (+/+) and CC (HAB-typical homozygotes, n = 67), (+/−) and CT (heterozygotes, n = 142), or (−/−) and TT (LAB-typical homozygotes, n = 49). (A) % time spent on the open arms of the elevated plus-maze (EPM) and (B) total distance traveled in the open field (OF). LAB-typical homozygotes were less anxious, independent of locomotor activity. Data are shown as means+SEM; * p<0.05.

To further test their functional involvement, the *Avp*-related polymorphisms were analyzed in a freely-segregating F2 panel (n = 508). In a total of 258 male mice with HAB grandmothers, 49 animals were identified as carrying the LAB-typical polymorphisms, i.e. the 12 bp deletion and the C(40)T in the combination −/− and TT, 67 animals the HAB-typical +/+ and CC and 142 animals the heterozygous +/− and CT genotypes. In all F2 animals, the allele distribution roughly reflected a 1∶2∶1 ratio (Table 6). If these polymorphisms have an impact on anxiety-related behavior, then homozygous −/− and TT F2 animals should behave less anxious than their +/+ and CC counterparts. Indeed, a co-segregation could be detected with −/− and TT mice being significantly less anxious on the EPM (p<0.05; Fig. 8A). Importantly, this association of the genetic polymorphisms with anxiety levels was independent of locomotor activity, measured in the open field (Fig. 8B). In the remaining 250 F2 male mice with LAB grandmothers, no association was detectable (data not shown).

**Table 6:** Allele frequency in a freely-segregating F2 panel (HABxLAB intercross offspring, n = 508) for the SNP in the *Avp* signal peptide and the strictly linked promoter deletion.

Locus (40)	Frequency in NAB	Δ(−2191–2180)
CC	25.9%	+/+
CT	51.9%	+/−
TT	23.2%	−/−

## Discussion

In this study, we identified polymorphisms in the *Avp* gene underlying a decreased expression of *Avp* mRNA in the hypothalamus of LAB animals under basal conditions compared to HAB, NAB and F1 mice, irrespective of gender. Both correlative and associative evidence indicates that the hypomorphic *Avp* allele causally contributes to the locomotion-independent reduction of anxiety-related behavior typical of LAB mice.

Importantly, differences in *Avp* expression were not accompanied by changes in V1a and V1b receptors that could confound the analysis of the behavioral involvement of AVP in these lines. Several groups independently reported an association of the V1a receptor with anxiety-related behavior. V1a receptor knockout mice, although at times providing inconsistent results, showed impaired social interaction, social recognition and reduced anxiety-related behavior [38]–[40]. Furthermore, V1a antisense RNA targeting the septum made rats less anxious [21]. Similarly, V1b antagonism was found to reduce anxiety- and depression-like behaviors [41], [42]. In the present study, expression profiling in combination with qPCR and receptor autoradiography revealed no V1a/b-related difference between HAB and LAB mice in a variety of anxiety-associated brain regions, suggesting that line-specific divergences in behavior are primarily due to events upstream of the receptor, i.e. differential expression and release of AVP.

Despite the high sequence homology of the evolutionary related and tail-to-tail oriented *Avp* and *Oxt* genes that are separated by 4 kbp, the so-called intergenic region [43], and despite the anxiolytic potency of the central OXT system [44], [45], differences in gene expression were only restricted to the former. This is consistent with the idea that our bidirectional breeding protocol selects for genetic variation in specific anxiety-related signaling pathways rather than disrupting hypothalamic functions in general.

To identify the molecular mechanisms underlying the reduced *Avp* expression, we sequenced the *Avp*-coding region and the surrounding structures. Nine polymorphic loci were identified reliably differing between HAB and LAB mice; all structural parts, the upstream promoter, the gene locus and the downstream enhancer region, contained three polymorphisms each. How may the polymorphisms in the promoter region translate into different expression profiles and phenotypes? While the C(−1422)T SNP is not located in a known transcription factor binding site, the T(−2521)C SNP and the Δ(−2180–2191) deletion are located directly in the center of a binding site for nuclear factor 1 (NF-1). This well-described transcription factor in the brain has the potential to enhance or impair gene expression, depending on the concomitance of a C/EBP binding site [46]–[49]. At the Δ(−2180–2191) deletion site, the binding of NF-1 would be impaired. The T(−2521)C SNP could enhance binding, but has no active concomitant C/EBP site, thus ultimately contributing to reduced gene expression. Thus, both polymorphic sites are likely to lead to an impaired *Avp* expression in mice bearing the LAB-specific DNA sequence.

While there were no polymorphisms identified in the non-coding (untranslated region and intronic) sequences, all three SNPs in the gene locus were located in the coding sequence. Two of these are silent mutations (A(1431)G and T(1527)C), and the third one (C(40)T) causes a substitution of alanine to valine in the third amino acid of the AVP signal peptide. As already reported by Keßler *et al.* [25], this genetic marker co-segregated in an F2 panel with symptoms of central diabetes insipidus in LAB mice and, partially, with anxiety-related behavior, further confirming the strict linkage between the promoter deletion Δ(−2180–2191) and C(40)T.

Also SNPs in the downstream enhancer region can have an impact on expression. There is a repetition of motifs from the 178-bp region between +370 bp and +480 bp [43], where two further SNPs have been identified. Analysis of this region resulted in the identification of a binding site for c-Ets-2 near a binding site of C/EBPbeta [48]. In the center of the c-Ets-2 binding site, the A(+399)G polymorphism would allow for an enhanced transcription rate in the HAB-specific but not the LAB-specific DNA sequence. Finally, even polymorphisms in the coding region are likely to contribute to lower *Avp* mRNA content in LAB mice by negatively influencing mRNA secondary structure and stability [50].

Still, we had to make sure that *Avp* under-expression in LAB mice is definitely due to the described genetic polymorphisms rather than to differences in other variables, including epistatic gene-gene interactions or synaptic input to the hypothalamic nuclei, likely to differ between the lines. Therefore, to eliminate the enormous complexity of the intact organism, we decided to use allele-specific transcription analysis, which had already been successfully applied in HAB/LAB rats [18]. By cross-mating HAB and LAB mice, we produced heterozygous F1 animals that host both HAB and LAB line-specific alleles in each cell. The analysis revealed a strongly decreased expression of *Avp* but not *Oxt* by the LAB-specific allele compared to the HAB-specific one, thus further confirming that line-specific polymorphisms are causally related to line-specific expression profiles of *Avp* and, consequently, the phenotype of trait anxiety.

However, while AVP expression is clearly under genetic control, the regulation of anxiety-related behavior is more complex. Compounding the difficulties associated with detecting multiple small or moderate genetic effects, the latter operate via subtle interactions with each other and the environment, thus further masking the functional significance of given polymorphisms as described here. Therefore, we used different approaches to examine the possible contribution of the hypomorphic allele-driven AVP deficit to trait anxiety, including correlational analyses in HAB/F1/LAB mice and association studies in F2 animals. Importantly, experimental crosses such as an F2 intercross are not subject to allelic association between genes on different chromosomes, thus eliminating spurious associations between markers and phenotypes [51]. Indeed, in addition to correlative evidence, a significant, though moderate association between the promoter deletion and the C(40)T SNP and locomotion-independent anxiety-related behavior was demonstrated in a freely-segregating F2 panel, indicative of a causal contribution to the phenotype. It is of note in this context that these F2 animals were assigned according to their *Avp*-related genotype, while all the other (anxiety-relevant) genes freely segregated, explaining why differences in anxiety-related behavior are rather modest (Fig. 8A) compared to those seen in Fig. 1A.

As mentioned before, the individual phenotype is known to be shaped by phenomena that are or are not mediated by sequence variations in DNA, i.e. inheritance is both genetic and environmental, with the latter primarily relying on the quality of mother-infant interaction [52]. To reduce possible influences of different epigenetic effects, deriving from the fact that half of the F2 had LAB and the other half HAB grandmothers, split calculations according to the grandmaternal background were performed for associations. Indeed, a significant association at the *Avp* locus was found for F2 mice with HAB grandmothers. Thus, while the polymorphisms identified in the *Avp* gene are unlikely to generally cause hypo-anxiety, their variation may contribute to the severity of the phenotype, further confirming the molecular basis of many small or moderate genetic effects giving rise to trait anxiety. In other words, while the link between genes, the brain and behavior remains weak, variation in the *Avp* locus may be one contributor, among many others, to trait anxiety. Several dozen quantitative trait loci, containing one gene or several genes, may be involved in anxiety regulation, and most of them are yet to be identified [53], [54].

Further studies will focus on the importance of the (grand)parental background for anxiety-related behavior, as genes regulated by (grand)parent-specific epigenetic modifications can lead to monoallelic gene expression [55], [56] or are simply X- or Y-linked. As there is a higher prevalence of anxiety and depression in women than in men [57], the (grand)parental influence on anxiety-related behavior, as revealed in our F2 panel, might also be due to a modulating effect of a sex-specific locus. HAB/LAB mice might thus represent a model to further investigate the complex pattern of genetic vs. epigenetic inheritance.

As LAB mice exhibit a deficit not only in AVP precursor processing and neuropeptide release from both dendrites and axon terminals [25], but also in *Avp* expression, additive effects at multiple levels are likely to produce a more or less “global” deficit in bioactive AVP. Remarkably, recent studies in Brattleboro rats and humans confirmed that an AVP deficit may be accompanied by symptoms of central diabetes insipidus, reduced anxiety-related or attenuated depression-like behavior [58] and signs of diminished agoraphobia [15], respectively, suggesting complementary inter-species genetics.

Determining the most common *Avp*-related genotypes in the NAB line revealed that the HAB-specific polymorphisms represent the most common genetic variant. More than 70% of the NAB mice were found to be homozygous for the HAB-specific sequence, whereas less than 1% carried the LAB-specific allele homozygously. Nevertheless, the respective distribution is in Hardy-Weinberg equilibrium, although with the LAB-specific allele at a decreased frequency. The fact that the NAB line's most common genotype for the *Avp* gene and surrounding sequence fully corresponds to the HAB-specific sequence helps to explain the often observed discrepancy between NAB and F1 mice, both of which are often used as controls. In contrast to strictly intermediate F1, NAB mice displayed in about 70% of all animals a HAB-like *Avp* expression and depression-like behavior in the tail-suspension test (Fig. 1B, D, E and [24]), which has also been demonstrated recently in an unselected CD1 population [59], thus further emphasizing the functional implications of centrally released AVP.

The application of similar breeding strategies in two different species (*Rattus norvegicus* and *Mus musculus*) resulted in similar findings concerning the identification of polymorphisms in and around the *Avp* gene and both the expression and release patterns of AVP. This was shown to be likely involved in mediating anxiety-related behavior. While these results cannot entirely exclude an effect of genetic drift, they strongly point to an evolutionary conserved dynamic role of AVP along a continuum, with hypomorphic *Avp* alleles finally contributing to low anxiety- and possibly depression-like behaviors and hypermorphic alleles contributing to hyper-anxiety. Together with human studies [23], [60], they do not only ensure the construct validity of the HAB/LAB model, but additionally provide a strong impetus for *Avp* expression as a potential biomarker and therapeutic target in psychopathology.
